# Coronavirus disease 2019 (COVID-19) mRNA vaccine and the risk of myocarditis: An increasing concern

**DOI:** 10.1017/ash.2021.209

**Published:** 2021-11-26

**Authors:** Omer Ahmed Shaikh, Priyanka Mohan Lal, Anmol Mohan, Um-Ul- Wara, Ana Carla dos Santos Costa, Shoaib Ahmad, Mohammad Yasir Essar

**Affiliations:** 1 Ziauddin Medical University, Karachi, Pakistan; 2 Karachi Medical & Dental College, Karachi, Pakistan; 3 Federal University of Bahia, Salvador, Bahia, Brazil; 4 Faisalabad Medical University, Faisalabad, Pakistan; 5 Kabul University of Medical Sciences, Kabul, Afghanistan

*To the Editor*—The coronavirus disease 2019 (COVID-19) pandemic has resulted in massive disruption across the world, putting additional pressure on health care. As the virus continues to spread, this health crisis poses further challenges to the healthcare system. As of June 20, 2021, the global coronavirus disease-2019 (COVID-19) pandemic has resulted in ∼179,075,360 cases and 3,877,769 deaths.^1^ To combat this novel virus, 3 COVID-19 vaccines were approved by the FDA: Pfizer-BioNTech COVID-19, Moderna, and Janssen.^2^ As of June 20, 2021, at least 1 dose of a COVID-19 vaccine has been administered to 21.5% of the world’s population. Globally, 2.6 billion doses have been administered, with 36.1 million doses administered every day.^3^

On December 11, 2020, the US Food and Drug Administration (FDA) granted an emergency use authorization (EUA) for the Pfizer-BioNTech COVID-19 mRNA vaccine for all individuals aged ≥16 years. The FDA revised the EUA for this vaccine on May 10, 2021, to include children aged 12 and up. The Pfizer vaccine is currently the only vaccine approved for use in children aged 12–17 years.^4^ According to the Centers for Disease Control and Prevention (CDC), almost 7 million teenagers and preteens (aged 12–17 years) in the United States have received at least 1 dose of the COVID-19 vaccine.^5^ The COVID-19 vaccine trials only involved a small number of children due to the urgent need for vaccination and the rapidly spreading nature of the virus. It is possible that rare but serious adverse events went unnoticed in this population.^4^

Vaccines have been linked to a variety of side effects, including injection site pain, fatigue, headache, muscle soreness, chills, joint pain, and fever. Myocarditis is a rare vaccination adverse event, especially after smallpox vaccination.^4^ Since April 20, 2021, reports of myocarditis and pericarditis after mRNA COVID-19 vaccination (Pfizer-BioNTech and Moderna) to the Vaccine Adverse Event Reporting System (VAERS) have surged in the United States, particularly among teenagers and young people.^6^ This serious adverse effect might contribute to vaccine hesitancy even if the side effect is rare.

Currently, officials from the Department of Health and Human Services have confirmed 226 occurrences of myocarditis or pericarditis in patients aged ≤30 years who received the mRNA COVID-19 vaccine, and another 250 cases are under investigation.^7^

Myocarditis is a host’s immune response on the myocardial tissue causing cardiac injury and impaired function without any obstructive cause. It can occur for various reasons, including viral infections, pathogens, and hypersensitive drug reactions. The presenting complaint varies among individuals and commonly includes mild dyspnea, fatigue, chest pain, and symptoms of acute-onset heart failure.^8,9^ The blood work of such patients shows elevated levels of inflammatory markers and cardiac enzymes. Usually the electrocardiogram (ECG) also confirms the diagnosis by ST-segment elevation and PR-segment depression.^8^ More research is needed to understand this rare condition.

In the Pfizer-BioNTech clinical studies, younger research participants showed greater systemic reactogenicity and immunogenicity after receiving the mRNA vaccine. Myocarditis or myopericarditis could be another unusual adverse event linked to systemic reactogenicity; however, no direct link between the Pfizer vaccine and myopericarditis has been demonstrated at this time.^4^

Additional study in this age group is needed to confirm the correlation of myocarditis to COVID-19 vaccination. Until this relation is investigated, all physicians and emergency department staff should be advised to consider myocarditis in their differentials of adolescents presenting with acute chest pain, shortness of breath, radiating pain to the arms and paresthesia, following COVID-19 vaccination. All confirmed cases of myocarditis in suspected individuals should be promptly reported to the Vaccine Adverse Events Reporting System (VAERS) for active monitoring and further investigation.

Considering the high morbidity rate associated with severe acute respiratory coronavirus virus 2 (SARS-CoV-2) infectivity, the benefits of the vaccine outweigh the risks associated with it; thus, the American Heart Association strongly recommends getting vaccinated as approved by the CDC and FDA, as an antecedent to a COVID-free world.^10^
